# Quantification of the *In Vitro* Radiosensitivity of Mung Bean Sprout Elongation to 6MV X-Ray: A Revised Target Model Study

**DOI:** 10.1371/journal.pone.0128384

**Published:** 2015-06-08

**Authors:** Tzu Hwei Wang, Samrit Kittipayak, Yu Ting Lin, Cheng Hsun Lin, Lung Kwang Pan

**Affiliations:** 1 Department of Radiotherapy Oncology, Buddhist Tzu Chi General Hospital, Taichung Branch, Taichung, 427, Taiwan; 2 Graduate Institute of Radiological Science, Central Taiwan University of Science and Technology Takun, Taichung, 406, Taiwan; 3 Department of Radiology, China Medical University Beigang Hospital, Yunlin, 651, Taiwan; University of South Alabama Mitchell Cancer Institute, UNITED STATES

## Abstract

In this study, a revised target model for quantifying the *in vitro* radiosensitivity of mung bean sprout elongation to 6-MV X-rays was developed. The revised target model, which incorporated the Poisson prediction for a low probability of success, provided theoretical estimates that were highly consistent with the actual data measured in this study. The revised target model correlated different *in vitro* radiosensitivities to various effective target volumes and was successfully confirmed by exposing mung beans in various elongation states to various doses of 6-MV X-rays. For the experiment, 5,000 fresh mung beans were randomly distributed into 100 petri dishes, which were randomly divided into ten groups. Each group received an initial watering at a different time point prior to X-ray exposure, resulting in different effective target volumes. The bean sprouts were measured 70 hr after X-ray exposure, and the average length of the bean sprouts in each group was recorded as an index of the mung bean *in vitro* radiosensitivity. Mung beans that received an initial watering either six or sixteen hours before X-ray exposure had the shortest sprout length, indicating that the maximum effective target volume was formed within that specific time period. The revised target model could be also expanded to interpret the “*two-hit*” model of target theory, although the experimental data supported the “*one-hit*” model. If the “*two-hit*” model was sustained, theoretically, the target size would be 2.14 times larger than its original size to produce the same results.

## Introduction

A target model is highly effective for determining the probability of cell radiosensitivity. Many studies have incorporated clinical findings of radiation biology studies by adopting the target model despite the complex relationship between a given dose transferred to the specific target and the probability of cell radiosensitivity. By using the cell survival function, Aardweg et al. interpreted the effect of split-dose irradiation on primary and metastatic uveal melanoma cell lines [1]. Brenner et al. illustrated the survival probability of tumor cells based on a simplified exponential function of a linear-quadratic model [2]. While also using the linear-quadratic model, Balagamwala et al. described the single or double hit of the cell size target, and claimed that the cell had sublethal damage if the *two hit* model sustained. Additionally, the G2 and M phases of the cell cycle had the highest radiosensitivity owing to their comparatively large sizes, explaining why any given dose of radiation would selectively kill a certain population of cells leaving a cell cycle-synchronous radioresistant population [3]. Hsieh et al. proposed a revised linear-quadratic form of an exponential function to interpret the heavy ion interaction onto the cell-killing effect of the boron neutron capture therapy and reached satisfactory correlations between practical measurement and theoretical estimation [4]. Accordingly, an ionizing particle striking a macromolecular structure in a cell may initiate a cell mutation, which can be defined as irreversible damage caused by a direct or indirect “*hit*” from a radioactive particle. Accordingly, a large macromolecular structure has a high radiosensitivity under a given intensity of radioactive particles. Despite the potential complexity of biological mechanisms underlying radiation-induced cell mutations on the molecular scale, the relative quantification of sprout elongation in mung beans in different sprout elongation states can still be elucidated.

In this study, a complete mathematical description of the *in vitro* radiosensitivity of mung bean sprout elongation was developed by combining a revised “*one-hit*” target model with the Poisson prediction of low probability of success. The target model was confirmed by exposing 100 petri dishes of mung beans in various states of sprout elongation to various doses of 6-MV X-rays generated and guided by a medical 6-MV linear accelerator (LINAC). The mung beans showed varying sensitivities to radiation; the sensitivities were expressed in terms of varying effective target volumes. A high *in vitro* radiosensitivity implied a large effective target volume and was associated with a low sprout elongation for a given exposure.

The *in vitro* radiosensitivity of sprout elongation was significantly related to both the time at which the initial watering before X-ray exposure took place and the linear *“no hit”* curve predicted by the revised target model. The interpretation of the results obtained using this revised target model with respect to the *in vitro* radiosensitivity of sprout elongation is discussed based on the experimental results obtained in this study.

## Materials and Methods

### Revised Target Model

In this study, mung beans were exposed to X-ray doses. The radiation dose provides deposited energy, which is transported into the specific volume of a target. Therefore, to deposit a particular amount of energy into a target, the number of high linear energy transfer (LET) radioactive particles such as alpha or heavy charged particles must be much lower than the number of low LET particles (i.e. beta particles, X-rays or gamma-rays) striking the target [5]. Many bullets (where a large particle is considered a heavy bullet, and a beta particle, X-ray or gamma-ray is considered a light bullet) are also associated with a high probability of hitting a target and causing cell mutation. The revised target model assumes that all energy absorbed by a specific target can be converted into a dimensionless *“hit”*. The particles with a high LET can create many *“hits”* in a single interaction because a high-LET particle, *i*.*e*., a heavy bullet, can transfer substantial energy to a specific target volume. In contrast, particles with a low LET must be combined to perform a complete *“hit”* because little energy is transferred to the target from a low-LET particle, *i*.*e*., a light bullet. Therefore, the *“hit”* can be defined by Eq 1:

$$hit=\frac{V\left[{\text{m}}^{3}\right]\rho \left[{\text{kgm}}^{-3}\right]D\left[{\text{Jkg}}^{-1}\right]}{\varepsilon \left[\text{J}\right]}$$ (1)

where *“hit”* is a hypothesis and the average number of *“hits”*, andε(J) is the mean energy absorbed by a specific target to make a *“hit”* which is defined as 1,000 J in this algorithm. Additionally, V (m^3^) is the effective target volume, D (Jkg^-1^) is the absorbed dose, and ρ (kgm^-3^) is the density of the target, which is 1,000 kgm^-3^ (*i*.*e*., the density of water). The product *V*ρ*D* is the total deposited energy absorbed by the specific target volume, and its mean value (*V*ρ*D/*ε) can be defined as the average number of *“hits”* onto the target. The effective target volume V can be manipulated by changing the quantity of assigned ε and correlates with the ε value. However, since the ε value is easily offset by water density (ρ = 1,000 kgm^-3^), to simplify the calculation of “*hit”* (*hit = VD*) (cf. Eq 1), the assigned ε(equals 1,000 (J) in this work) is preferable in the normalized process of the revised target model; furthermore, the definition ofεhas its unique feature in retaining the dimensionless of *“hit”*. Restated, the energy absorbed by a specific target can be transformed into the receiving “*hit”* by the target. Eventually, the absorption of high energy also becomes multiple “*hits”* to the target in this revised target model. However, the small size of the target makes a successful *“hit”* in the molecular range (~100 nm) difficult. The low probability of a successful radiation-induced *“hit”* in each trial can be estimated, and the product of the probability of success as well as the number of trials gives the Poisson distribution (Eq 2) [6]

$$P(n)=\frac{{\bar{X}}^{n}\cdot e^{-\bar{x}}}{n!}{}^{}or{}^{}\frac{{\left(\frac{V\rho D}{\varepsilon }\right)}^{n}\cdot e^{-\frac{V\rho D}{\varepsilon }}}{n!};$$ (2)

where $\bar{X}$ is the expected value of the Poisson distribution and the mean number of “*hits”* in the specific target. The normalized summation of all of the mean numbers of “*hits”* recorded on the target equals unity and is expanded as a Taylor expansion in Eq 3.

$$\begin{array}{l} \sum_{n=0}^{\infty }P(n)\equiv 1=e^{-\frac{V\rho D}{\varepsilon }}\cdot e^{\frac{V\rho D}{\varepsilon }}\;\\ \;=e^{-\frac{V\rho D}{\varepsilon }}\left(1+\frac{V\rho D}{\varepsilon }+\frac{1}{2!}{\left(\frac{V\rho D}{\varepsilon }\right)}^{2}+\frac{1}{3!}{\left(\frac{V\rho D}{\varepsilon }\right)}^{3}+\frac{1}{4!}{\left(\frac{V\rho D}{\varepsilon }\right)}^{4}+....\right);\\ e^{-\frac{V\rho D}{\varepsilon }}\cdot 1\to no\overset{}{}hit;\\ e^{-\frac{V\rho D}{\varepsilon }}\cdot (1+\frac{V\rho D}{\varepsilon })\to no+one\underset{}{}hit;\\ e^{-\frac{V\rho D}{\varepsilon }}\cdot [1+\frac{V\rho D}{\varepsilon }+\frac{1}{2!}{\left(\frac{V\rho D}{\varepsilon }\right)}^{2}]\to no+one+two\underset{}{}hit; \end{array}$$ (3)

According to Eq 3, *“no hit”* means that the radioactive particle may still deposit some energy in the target. Since this energy is insufficient to generate a complete *“hit”*, mutation is minimal. The term *“no+one hit”* denotes the probability that the target is hit by a particle either one time or not at all. Restated, *“no hit”* represents the probability of target function assuming a single complete *“hit”* is sufficient to mutate a target. Therefore, a particle with a high LET can cause many “*hits”*, whereas many particles with a low LET are needed to cause a complete “*hit”*.

Since the energy generated by either *“no hit”* or *“one hit”* by radioactive particles is insufficient for mutation, the mung bean sprout can withstand a single *“hit”* and still elongate normally if the predicted bean mutation is consistent with the calculated *“no+one hit”* probability. Additionally, *“no+one+two hit”* indicates that the mung bean sprout undergoes mutation only when it has received three *“hits”*, resulting in bean cell elongation rate that is the sum of the probabilities of *“no hit”*, *“one hit”* and *“two hits”*. For further study of the use of the revised target model in determining the *in vitro* radiosensitivity of an actual cell, the effective target volume of the specific cell is determined by increasing the radioactive dose. When the sprout elongation rate is reduced to 37%, the effective target volume is given by Eq 4 (Eqs 1 and 3). Restated, the product of the effective target volume V and (*ρD*_*37%*_/ε) equals exactly one in this revised target model. Additionally, 1 Gy (Jkg^-1^) equals 6.25×10^18^ (eVkg^-1^] in this calculation because 1 eV equals 1.6×10^–19^ (J].

$$\begin{array}{l} e^{-(\frac{V\rho D_{37\%}}{\varepsilon })}=e^{-1}=0.37;\\ (\frac{V\rho D_{37\%}}{\varepsilon })=\text{1;}{}_{}\text{Thus}\\ {\text{effective target volume (V) [m}}^{3}\text{]= }\frac{1}{\;(\frac{\rho D_{{}_{37\%}}}{\varepsilon })}\; \end{array}$$ (4)

Notably, the effective target volume is inversely proportional to the measured (*ρD*_*37%*_ */*ε). A large effective target volume corresponds to a low D_37%_ value, because a large target volume accumulates a lethal dose more quickly than a small target volume. A sprout that can still elongate under a high dose of radiation has a small effective target volume for a given dose. Restated, a small target structure is associated with a high sprout elongation probability. The exact amount of εis difficult to evaluate practically with this algorithm; however, the reciprocal of (*ρD*_*37%*_ */*ε) can be easily derived from the evaluation of the effective target volume, V (Eq 4). Accordingly, the size of the effective target volume implies exactly the probability of receiving a *“hit”* from the radiosensitivity viewpoint.

### Sample Preparation

Mung bean, also known as green bean, is the seed of *Vigna radiata* which is native to Bangladesh, India, and Pakistan. The major variety of mung bean planted in Taiwan is berken. The split bean is known as moong dal, Pesara, which is green with the husk, and yellow when dehusked. The beans are small, ovoid, and green. Mung beans are mainly cultivated in Taiwan, China, Thailand, and India, but also in hot and dry regions of southern Europe and the southern USA. Mung beans are tropical (or sub-tropical) crops, and require warm temperatures (optimally around 30–35°C). [7].

Five thousand fresh berken mung beans were randomly divided into 100 petri dishes (diameter, 9 cm), each containing 50 beans. The petri dishes were randomly divided into ten groups of ten dishes and labeled. The groups received an initial watering at different time points before X-ray exposure (see Table 1) and were then watered with 5 ml every two hours. The initial watering ensured that within a particular group, all the cells of the mung beans were in a synchronized sprout elongation state because watering mung beans shifted “resting” cells into the elongation process. The petri dishes were stored indoors at 25°C.

**Table 1:** The precise LINAC exposure plan for the ten groups of mung beans.

Group No.	Initial water time	Time before exposure	Total watering duration
**1**	D-1 day, 18:00	20 hr.	20 hr.
**2**	D-1 day, 20:00	18 hr.	18 hr.
**3**	D-1 day, 22:00	16 hr.	16 hr.
**4**	D day, 0:00	14 hr.	14 hr.
**5**	D day, 2:00	12 hr.	12 hr.
**6**	D day, 4:00	10 hr.	10 hr.
**7**	D day, 6:00	8 hr.	8 hr.
**8**	D day, 8:00	6 hr.	6 hr.
**9**	D day, 10:00	4 hr.	4 hr.
**10**	D day, 12:00	2 hr.	2 hr.
**LINAC Exposure**	D day, 14:00		

The watering schedule was set to let the mung beans in the different groups maintain in the different states, because the mung bean can be elongated by the given water.

### Exposure to LINAC

The petri dishes were transported to the Department of Radiology, Buddhist Tzu Chi General Hospital, Taichung branch, Taiwan, and exposed to 6-MV X-rays. The LINAC (Varian Trilogy model 3507) was set to a field size of 25×25 cm^2^ and a source-to-surface distance of 100 cm. The radiation doses were set to 1, 2, 5, 10, 20, 50, 100, 200 or 400 Gy. The LINAC head was rotated by 180°, and the X-rays were emitted from the bottom of the treatment couch toward the top. The exposed petri dishes were placed between two 20-mm-thick solid polystyrene plates to ensure uniform exposure to the radiation field. For each radiation dose, one petri dish from each of the ten groups was used. Because the mung bean groups received an initial watering at different times, the sprout elongation of the exposed mung beans was related to either the duration of watering or the X-ray dose. All of the data for the exposed mung beans were normalized to controls that were planted and watered under the same conditions.

### Samples Post-Processing

**Fig 1:**
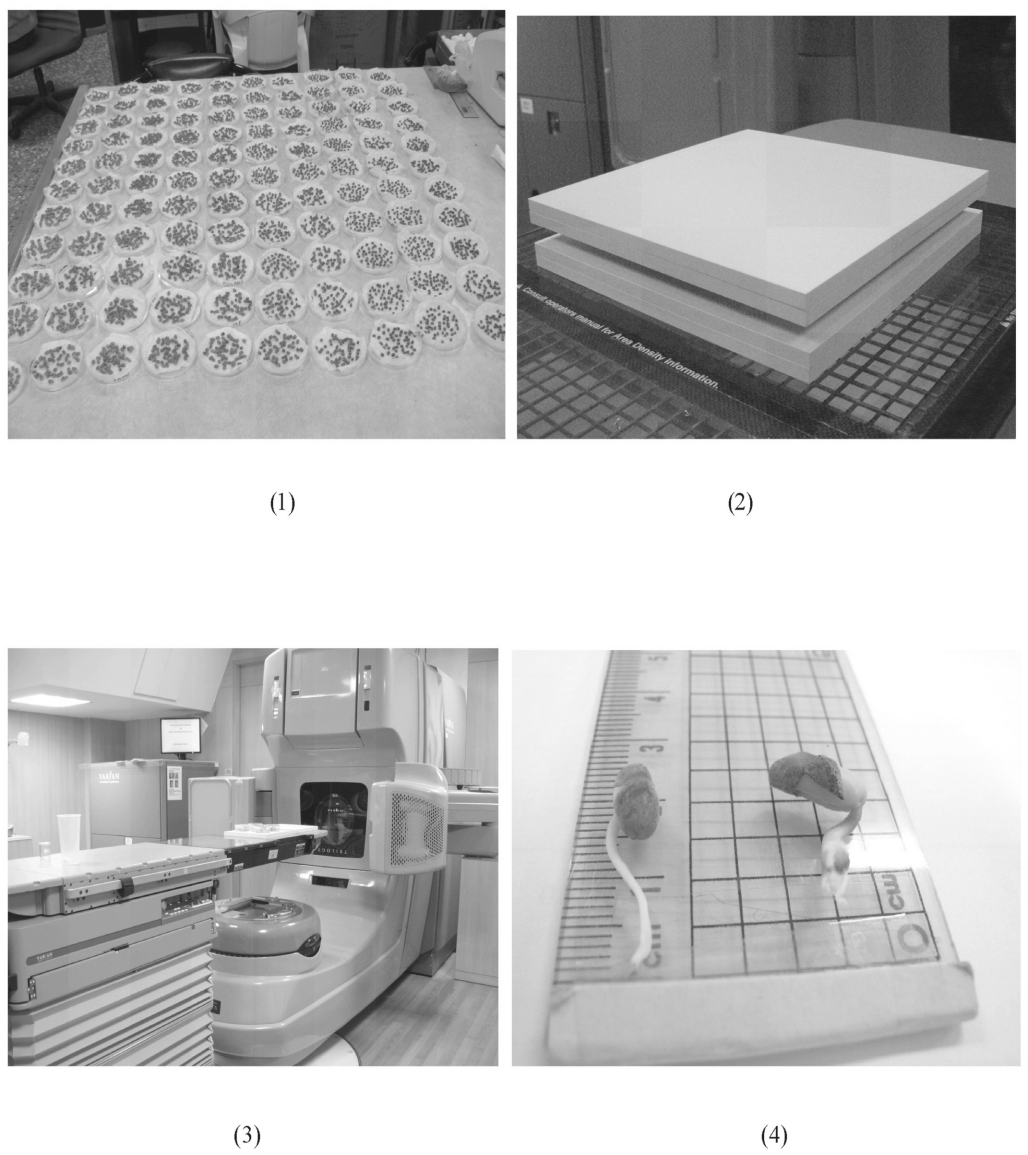
The real situation of mung bean inside the petri dishes (1) during watering, (2) between two 20 mm-thick solid polystyrene plates, (3) exposed to 6-MV LINAC with the head of LINAC placed bottom-down and the X-rays emitted from the bottom toward the top, and (4) during measurement with a ruler to within 1 mm.

The mung beans were kept indoors and received 5 ml of water every two to four hours for 70 hours to ensure growth of the bean sprout. In each dish, the lengths of the sprouts were measured. Using Eq 5, which is revised version of an equation from Preuss *et al*. [8] and Scott *et al*. [9], an index of the cell elongation rate after X-ray exposure was obtained.

$$\text{sprout}\;\text{elongation}\;\text{rate}=\frac{\bar{L_{ij}}}{\bar{L_{i0}}}\times 100\%$$ (5)

where *L*_*ij*_ and *L*_*io*_ are the average lengths of the sprouts after the *jth* Gy dose and no dose (control), respectively in the *ith* group of Petri dishes. A high average length of bean sprouts can be considered to represent a high cell elongation rate and resistance to a high X-ray dose. Fig 1 presents the petri dishes containing mung beans (1) during watering, (2) between two 20-mm-thick solid polystyrene plates, (3) exposed to 6-MV LINAC with the head of the LINAC placed bottom-down and the X-rays emitted from the bottom toward the top, and (4) during measurement with a ruler to within 1 mm.

## Results

### Sprout Elongation Rate

**Fig 2:**
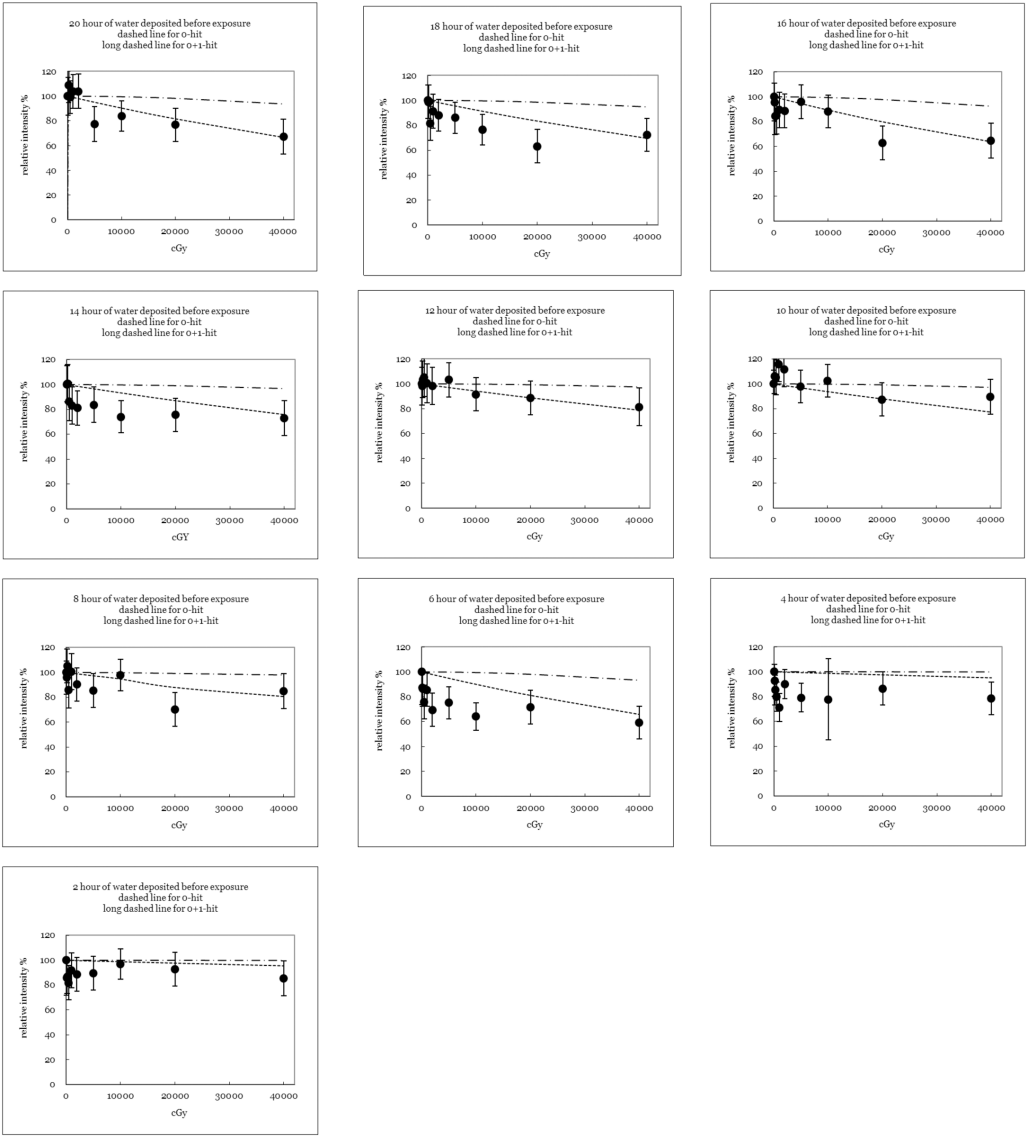
The precise plot of the normalized mung bean cell elongation rate in every group. The data are quoted from Table 2. The solid circles plot the normalized results for exposed bean sprouts; the dashed and long-dashed lines plot theoretical estimates under the *“no hit”* and *“no+one hit”* scenario, respectively, made using the revised target model.

Table 2 shows the normalized sprout elongation rates for the ten mung bean groups (Eq 5). The control group of mung beans that was not exposed to X-rays exhibited a 100% cell elongation rate, which is consistent with that predicted by Eq 5. However, in some cases, the average length of the mung bean sprouts in the exposed petri dishes exhibited a high elongation rate exceeding 100%. This might be the widely data fluctuation in real sprout elongation. Fig 2 shows the normalized mung bean sprout elongation rate for group 1–10. The solid circles plot the normalized results for the exposed bean sprouts; the dashed and long-dashed lines plot the theoretical estimates obtained using the revised target model for the *“no hit”* and *“no+one hit”* conditions. In the estimation, “*V*” was first determined by either interpolation or extrapolation of the sprout elongation rate to obtain the (*ρD*_37%_ */*ε) value for each group (Eqs 3 and 4). The sprout elongation rates varied greatly among the groups at low X-ray doses (<100 Gy) and declined at high X-ray doses (100–400 Gy). Except for groups 2, 4, 8 and 9, which were partially consistent with the theoretical estimates, the results were highly consistent with the *“no hit”* scenario. In addition, an inappropriate derivation of D_37%_ from the practical evaluation may cause the deviation among data in the four groups. Either too high or too low of a quantity of the derived D_37%_ may significantly distort the effective target volume in calculating the elongation probability (Eq 4).

**Table 2:** The calculated results of the normalized sprout elongation rate (%) for the ten groups of mung beans (Eq 5 and Table 1).

Dose (Gy)	Normalized mung bean sprout elongation rate (%) in Group No.									
	1	2	3	4	5	6	7	8	9	10
**0.0**	100.0	100.0	100.0	100.0	100.0	100.0	100.0	100.0	100.0	100.0
**1.0**	99.9	98.7	95.8	100.6	98.3	124.9	95.8	87.1	93.0	99.9
**2.0**	108.9	98.9	84.4	100.7	103.6	106.3	105.1	86.3	85.3	108.9
**5.0**	99.1	81.6	84.9	86.2	105.3	105.3	85.8	75.8	79.9	99.1
**10.0**	103.7	91.1	89.4	83.1	100.4	115.9	100.8	85.6	71.2	103.7
**20.0**	104.0	88.0	88.8	81.2	98.4	111.8	90.4	69.5	90.0	104.0
**50.0**	77.3	86.1	96.0	83.8	103.3	98.0	85.6	75.2	79.1	77.3
**100.0**	83.9	76.4	88.3	74.2	91.7	102.5	97.9	64.1	77.7	83.9
**200.0**	76.7	63.2	63.0	75.6	88.7	87.5	70.3	71.6	86.5	76.7
**400.0**	67.3	72.2	64.8	73.0	81.5	89.6	85.0	59.3	78.5	67.3

### Critical Volume of Effective Target

**Fig 3:**
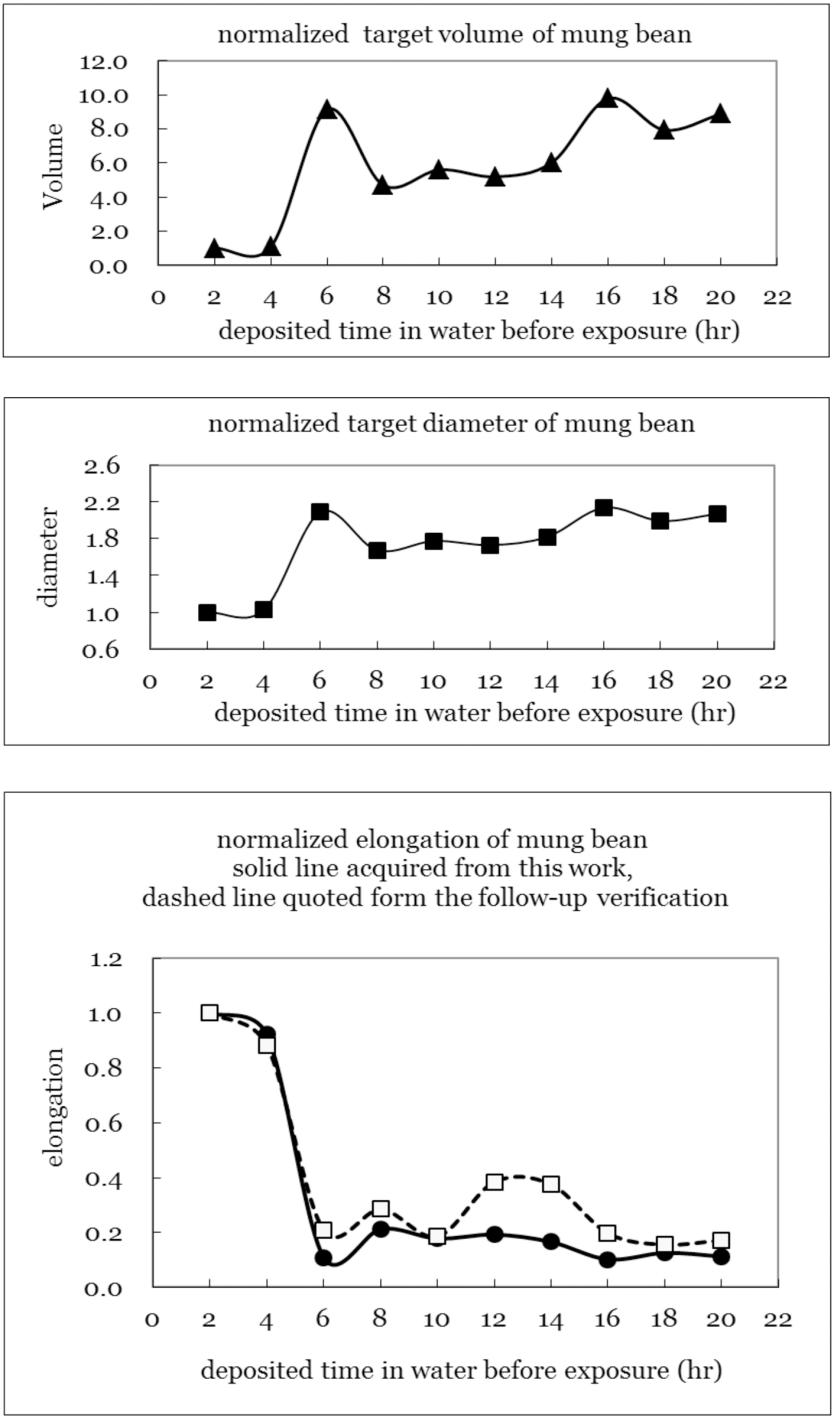
The analyzed effective target volumes, diameters and cell elongation rate in all groups. The plotted data were all normalized to the last group as was its first watering time 2 hours before the X-ray exposure (Table 1, group 10).

The critical volume of the mung bean cells in the various states was defined as the effective target volume. Additionally, the effective target volume and diameter were easily determined from the derived 1/(*ρD*_37%_ */*ε) value (Eq 4). The calculation of the diameters corresponding to the volumes assumed a spherical effective target volume (*V = (4/3)πr* ^*3*^). Fig 3 presents the calculated effective target volumes, diameters and sprout elongation rates for all of the groups. The plotted data were all normalized to group 10, which was first watered two hours before X-ray exposure (Table 1, group 10). The sprout elongation rate for each group was defined as the reciprocal of the effective target volume; thus, a large target volume corresponded to a low sprout elongation probability under a high X-ray dose. The sprout elongation rate of the mung beans reached its maximum during the initial stages of sprout elongation.

Because mung bean sprout growth requires a moist environment, the bean cells remained in a resting state until their moisture content reached the threshold for sprout elongation [10]. Within the first two-four hours after the initial watering, the moisture content in the mung bean cells increased from the original 10–15% to 30–45% and sprout elongation began (Fig 3) [11]. Accordingly, the critical volume of the effective target of mung beans also expanded and thus the average elongation rate decreased. The sprout elongation rate decreased rapidly after the initial stages (<4 hours; groups 9 and 10; the normalized average maintained around 1.0) and then exhibited small fluctuation (groups 1–8, the normalized averages varied between 0.1 and 0.2) (Table 1). Interpretation of the sprout elongation changes may partially be owing to that the mung bean cell falls in different phases of cell mitosis cycle. Correspondingly, G2 and M phases of the cell cycle have the highest radiosensitivity.

## Discussion

### Comparison of Diameters of Effective Target Volumes among Studies

Our revised target model was used to interpret the results of other studies of radiation-induced effects because it allows a correlation between target radiosensitivity and size. Table 3 shows the exact diameters of the effective target volume of all of the mung beans groups. The reference data are also provided for comparison. All of the diameters were calculated from the reported data using Eq 4, and the *D*_*37%*_ doses in each study were either interpolated or extrapolated from the obtained data. Any misinterpretation of the D_37%_ dose may cause the discrepancy between theoretical estimation and practical data as obtained in the groups 2, 4, 8, and 9 in this work (Fig 2). Additionally, the calculations for all of the diameters assumed the *“no hit”* scenario, *i*.*e*., only a single *“hit”* on a target mutates the target.

**Table 3:** The precise data of diameter of the mung bean effective target volume in all groups, and some referred data are also listed for comparison.

Reference No.	Target	Exposure source	*D*_*37%*_ (Gy)	Diameter of effective target volume (nm)
**[9]**	PC3	Ionizing radiation	2.60	49.0
	LNCaP		2.40	50.3
	CWR22R		3.30	45.2
**[12]**	FMDV	Gamma ray	2700	4.84
	RLV		570	8.12
	HSV		1120	6.49
**[13]**	Medical herb	Gamma ray	550	8.22
**[14]**	U-87 MG tumor cell	LINAC	6.00	37.1
**[15]**	PQ30	Gamma ray	321	9.84
	OG100		178	12.0
	IN99		20.3	24.7
	IN602		20.3	24.7
**[16]**	ME-180	Gamma ray	5.50	38.2
	HeLa-S3		6.00	37.1
**[17]**	Nipponbare	12C-ion	52.0	18.0
	Norin 1		36.0	20.4
	Sasanishiki		30.0	21.7
**[18]**	Curcuma alismatifolia	Gamma ray	28.0	22.2
**[19]**	6 Euphorbia pulcherrima cultivars	60Co Gamma ray	28.0–20.0	22.2–24.8
**This study**	Mung bean	6 MV X-ray by LINAC		
	Group 1		985	6.77
	Group 2		1102	6.52
	Group 3		893	6.99
	Group 4		1449	5.95
	Group 5		1684	5.66
	Group 6		1560	5.81
	Group 7		1863	5.47
	Group 8		957	6.83
	Group 9		8058	3.36
	Group 10		8740	3.27

The derived diameters of all reported data can be calculated according to Eq 4 and the *D*_*37%*_ dose in every work was either inter- or extrapolated from the reported data.

Table 3 clearly shows that the derived diameters of the effective target volumes vary widely from 3.27 nm to 50.3 nm and fall within the size range of DNA. Notably, a typical DNA double helix chain is 2.2 to 2.6 nm (10^–9^ m) wide, and one nucleotide unit is 0.33 nm long [20]. In living organisms, DNA usually appears as pairs of molecules held tightly together rather than a single molecule [21, 22]. The comparatively larger diameter of the effective target volume might still be accepted since an effective target can receive one *“hit”* either directly or indirectly. In a direct *“hit”*, ionization by a particle with a macromolecular target causes a direct mutation. In an indirect *“hit”* however, generation of a radical by intracellular water molecules reacting with the molecule causes a mutation [1]. Therefore, a derived effective target volume with a large diameter implies a high probability of an indirect *“hit”* breaking the molecular structure. In addition, the derived diameter of an effective target volume can be changed by the default ε (Eq 1, ε = 1,000 J). The calculation in this algorithm is eventually a relative quantification, although the exact quantity of εis still debatable in reality.

Nevertheless, a similar hypothesis can also be applied to neutron activation analysis. A radionuclide that is easily activated by a thermal neutron has a large thermal neutron activation cross-section. For example, a cobalt-59 nuclide has a large cross-section of 37 barns (37×10^-24^ cm^2^) [23]. The calculated diameter of cobalt-59 approximates 69 pm (69×10^-15^ m) although the actual diameter of this nuclide approximates 10 pm (1.3×59^1/3^ = 10.1 pm) [24]. Biomechanically, the diameter of the effective target volume correlates with radiosensitivity and can be interpreted consistently according to the revised target model. Therefore, the unusually large diameter of LNCaP cells (50.3 nm) [9], which causes *D*_*37%*_ fatal damage, occurs at only 2.40 Gy. Conversely, *D*_*37%*_ fatal damage in the FMDV cell [12] requires 2700 Gy because the diameter is only 4.84 nm. Kamba *et al*. used the heavy ^12^C ion to bombard three species of Japanese rice and then correlated the radiosensitivity of rice germination to various doses [17]. The definition of the sprout elongation rate used by Kamba *et al*. is similar to that used in the current study [Eq 5]. In addition, Preuss *et al*. also measured the length of the sprouts from uvh1 plant seeds exposed to high gamma ray doses [8]. However, the comparatively lower doses used by Kamba *et al*. may have been due the heavy ^12^C ion (heavy bullet) induced by either a high LET mechanism or by many indirect *“hits”* to the rice (*i*.*e*. large target size) (Table 3).

### One-Two-Hit Model

**Fig 4:**
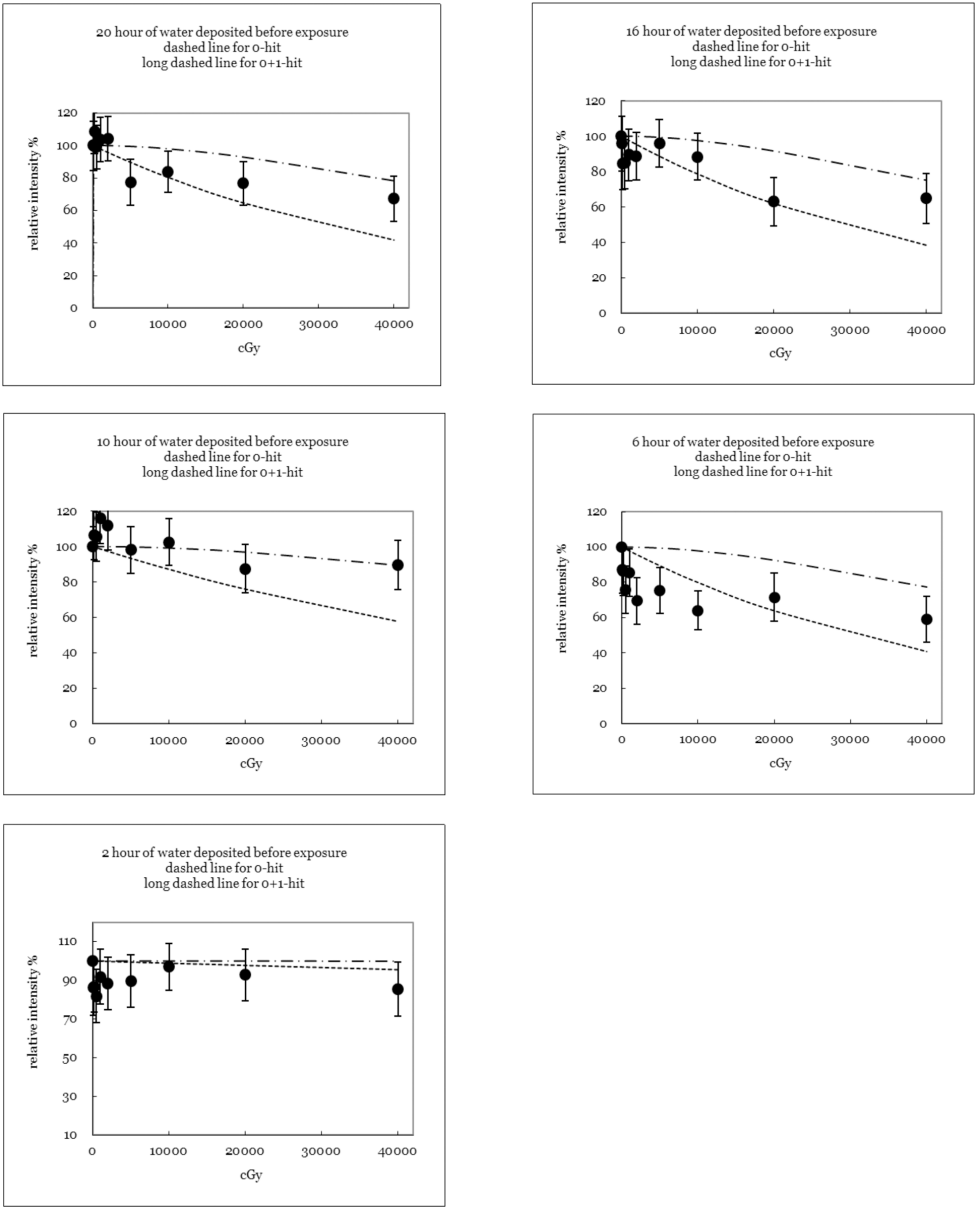
Five groups (1, 3, 5, 8, and 10) of experimental data (20, 16, 10, 6, and 2 hour of watering time before the X-ray exposure) were fitted to a revised *“no+one hit”* model in this work (cf. Fig 2).

The data in this study do not indicate that two *“hits”* are required for target mutation (Fig 2 groups (1)-(10)). The conventional methods of estimating sprout elongation rate using a target model are consistent with the hypothesis that one *“hit”* can cause only a mutation. Therefore, the sprout elongation rate is better estimated using the *“two-hit”* (*i*.*e*. *“no+one hit”* here) scenario in the micro-dosimetry to account the DNA double-helix-break damage. Additionally, Fig 4 shows that some theoretical assumptions are revised to fulfill the criteria of a *“two-hit”* model (cf. Fig 2, group 1, 3, 5, 8 and 10). Fig 4 clearly shows that the derived diameter of the effective target volume is 2.14 times larger than the original one derived from the *“one-hit”* model in this work. The enlargement coefficient is easily derived by applying Eq 6 because the specific target volume requires two *“hits”* to provide sufficient energy for mutation. The large effective target volume also implies that many biological reactions occur at the same intensity for a given dose.

$$e^{-\frac{V\rho D_{37\%}}{\varepsilon }}(1+\frac{V\rho D_{37\%}}{\varepsilon })=e^{-1}=0.37$$ (6)

In contrast, the data in Figs 2 and 4 do not support the *“two-hit”* scenario because (1) the empirically plotted curves barely match the *“two-hit”* model for any group and (2) the empirical data show no apparent shoulder effects for any group. One interpretation is that the derived diameter of the effective target volume exceeds the size of the actual DNA molecule. One *“hit”* from a radioactive particle can easily cause many irreversible mutations in DNA within a few nucleotide units (each of which is only 0.33 nm long). Therefore, a single *“hit”* from a radioactive particle can still fatally mutate a target that is in the size range of DNA (here, the derived diameter of the effective target volume is 3.27–6.99 nm).

The microscopic size range of DNA makes any investigation of DNA complicated. In contrast, sample diversity and uncertainty in evaluating the sprout elongation rate can be effectively suppressed in this study by averaging over a large number of samples and performing internal normalizations to combine the various groups of raw data. Thus, the revised target model with respect to the *in vitro* radiosensitivity of sprout elongation is successfully confirmed by the experimental results.

Furthermore, the target model adopted in most works is assumed to be a simple exponential function with a linear-quadratic form, *i*.*e*. exp(-αD-βD^2^), to obtain the correlation between survival probability and a given dose. Nevertheless, the ratio of α/β is the critical index in defining the shoulder effect of the two-hit model in order to verify the practical data [2–4]. However, the quantified parameters adopted in the revised target model significantly contributes to estimating the *two-hit* model by direct calculation; in addition, the inappropriate assumption is omitted when inspecting the ratio of α/β. Doing so greatly facilitates efforts to handle the radiobiological mechanism in order to interpret the radiosensitivity of a microscopic size target.
